# Myxinidin2 and myxinidin3 suppress inflammatory responses through STAT3 and MAPKs to promote wound healing

**DOI:** 10.18632/oncotarget.20908

**Published:** 2017-09-15

**Authors:** Hyo Mi Han, Sujin Ko, Min-Ju Cheong, Jeong Kyu Bang, Chang Ho Seo, Tudor Luchian, Yoonkyung Park

**Affiliations:** ^1^ Department of Biomedical Science, Chosun University, Gwangju, Korea; ^2^ Department of Life Science, Chosun University, Gwangju, Korea; ^3^ Division of Magnetic Resonance, Korea Basic Science Institute, Ochang, Korea; ^4^ Department of Bioinformatics, Kongju National University, Kongju, Korea; ^5^ Department of Physics, Alexandru I. Cuza University, Iasi, Romania; ^6^ Research Center for Proteinaceous Materials, Chosun University, Gwangju, Korea

**Keywords:** myxinidin, antimicrobial peptide, STAT3, MAPKs, wound healing

## Abstract

Skin wounds are continuously exposed to bacteria and can easily become infected. Infected wounds require antibiotic treatment, and infections caused by drug-resistant bacteria are an important public health problem. Antimicrobial peptides have broad-spectrum antibacterial activity, induce little or no drug resistance and may be suitable for treating skin infections caused by drug-resistant bacteria. We previously reported the design and function of myxinidin and myxinidin analogues. Here we showed that myxinidin2 and myxinidin3 exhibit antimicrobial and anti-biofilm activities against antibiotic-resistant *Staphylococcus aureus*, *Acinetobacter baumannii,* and *Pseudomonas aeruginosa* in high salt environments and in gelatin. Moreover, these peptides facilitated infected wound healing by decreasing inflammation through suppression of IL-6, IL-8, and TNF-α and regulation of downstream mediators such as STAT3, p38, JNK, and EGFR. In a mouse skin wound model infected with antibiotic-resistant bacteria, myxinidin2 and myxinidin3 eliminated the infection and enhanced wound healing. We therefore propose the use of these peptides for treating infected wounds and burns.

## INTRODUCTION

Bacterial infection of a skin wound can cause wound healing to be delayed and even cause a worsening of the wound. Most wound infections are caused by pathogenic bacteria such as *Staphylococcus aureus* [1], *Acinetobacter baumannii* [2], and *Pseudomonas aeruginosa* [3]. Some stains of these pathogens, which can also cause acute pneumonia, septicemia, and secondary infections in the brain or in other internal organs, are resistant to antibiotics in part because they form biofilms [4]. Primary skin infections stimulate inflammatory responses, characterized by calor, dolor, rubor, and tumor (the hallmarks of inflammation) [5]. Treatments for skin wounds are aimed at relieving the inflammation while enhancing immune system function through modulation of cytokines, transcription factors, and other proteins or genes [6]. In addition, efforts are being made to develop improved dressing materials for wounds, including hydrated biomaterials typically composed of polysaccharides such as gelatin. These biomaterials have numerous advantages in that they are biocompatible, biodegradable, and bacteriostatic, and they induce wound healing similar to that of natural tissues [7].

The inflammatory response in skin plays an essential role in the defense against pathogens. This response involves a complex interplay of cytokines and chemokines, such as the pro-inflammatory cytokines interleukin (IL)-8, tumor necrosis factor (TNF)-α and IL-6, as well as anti-inflammatory cytokines [8] and diverse inflammatory factors such as signal transducer and activator of transcription (STAT), mitogen activated protein kinase (MAPK), and nuclear factor kappa-light-chain-enhancer of activated B cells (NF-κB) [9]. TNF-α, IL-6, and IL-8 are the most active and well-characterized players during the pathogenesis of skin infections [10]. They control inflammatory responses through sequential phosphorylation of upstream kinases such as STAT3 [11], p38 [12], and Jun-amino-terminal kinase (JNK), leading to the activation of NF-κB, which promotes the transcription of several pro-inflammatory cytokines [13]. STAT3 secretion also induced through the activation of the epidermal growth factor receptor (EGFR), which is involved in diverse cell functions, including the regulation of cell proliferation, differentiation, survival, and motility, processes crucial to skin wound healing [14, 15]

In most organisms, antimicrobial peptides (AMPs) constitute a non-specific innate defense system against invading microbes [16]. Because of their broad-spectrum antimicrobial and anti-biofilm activity, their stability, and their low rates of resistance, AMPs are now being extensively studied. One such AMP, myxinidin, was originally isolated from hagfish epidermal mucus. It was then modified to develop two new smaller peptides, myxinidin2 and myxinidin3, which exhibit strong, selective antibacterial and anti-biofilm effects with no cytotoxicity in the tested mammalian cells [17]. AMPs such as myxinidin2 and myxinidin3 are a potential source of novel topical agents for the treatment of infected wounds [18].

In the present study, therefore, our aim was to assess the potential utility of myxinidin2 and myxinidin3 as agents for the treatment of wounded skin infected with antibiotic-resistant bacteria. This in part entailed testing the effects of these peptides on inflammatory cytokine production mediated via STAT3, p38, JNK, and MAPK signaling pathways, as well as NF-κB activity, in *S. aureus-*, *A. baumannii-*, and *P. aeruginosa*-infected human keratinocytes. We also assessed the effects of these peptides *in vivo* in a mouse model of drug-resistant bacterial skin infection. We anticipate our results will shed important new light on the use of these AMPs for the treatment of infected wounds.

## RESULTS

### Antimicrobial and bactericidal activity of myxinidin2 and myxinidin3 against drug-resistant bacterial strains

Myxinidin2 and myxinidin3 were evaluated for their antibiotic activity against one gram-positive strain (*S. aureus*) and two gram-negative species (*A. baumannii* and *P. aeruginosa*), including several antibiotic-resistant strains (Supplementary Figure 1). Myxinidin2 and myxinidin3 both exhibited good inhibitory or bactericidal activities against *S. aureus*, *A. baumannii* and *P. aeruginosa*, including the resistant strains. For example, myxinidin2 and myxinidin3 were effective against antibiotic-resistant *S. aureus* CCARM 3018, *A. baumannii* 719705, and *P. aeruginosa* 4076. The MICs against *S. aureus* CCARM 3018, *A. baumannii* 719705, and *P. aeruginosa* 4076 were, respectively, 25 μM, 12.5 μM, and 3.1 μM for myxinidin2 and 6.3 μM, 6.3 μM, and 1.6 μM for myxinidin3. The MBCs for myxinidin2 against the three strains were 50 μM, 12.5 μM, and 6.3 μM, and for myxinidin3 the MBCs were 6.3 μM, 6.3 μM, 6.3 μM. Thus, the MBCs ranged from 1-2 times the MIC. These AMPs also showed similar activity (concentration) against the other strains tested.

### Cytotoxicity of against HaCaT cells and their antibiotic efficacy against drug-resistant bacteria

**Figure 1:**
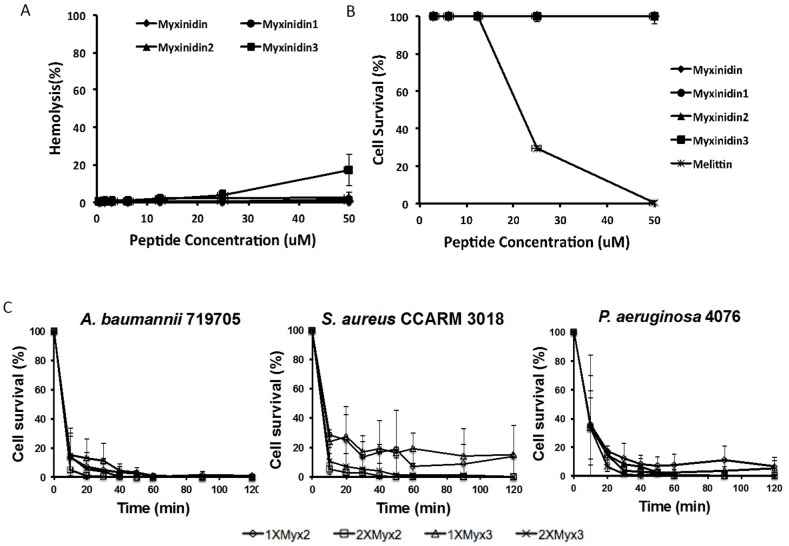
Antibiotic killing kinetics of myxinidin2 and myxinidin3 **(A)** Hemolytic activity of the indicated peptides against RBCs. Release of hemoglobin was measured at 414 nm. **(B)** Cytotoxicity activity of the peptides assessed using by MTT assays. Normal human keratinocytes were used to evaluate cytotoxicity. Measured was the absorbance at 570 and 650 nm. **(C)** Time-to-kill kinetics curve for the indicated peptides against *S. aureus* 3018, *A. baumannii* 719705 and *P. aeruginosa* 4076. Each experiment was performed in triplicate, and the value shown is the average of each experiment.

First, to assess the cytotoxicity of myxinidin2 and myxinidin3 against HaCaT cells, MTT assays were conducted using melittin as a positive control. At 50 μM, myxinidin2 and myxinidin3 showed no cytotoxicity, whereas melittin exhibited 70% toxicity at 25 μM. Then in hemolysis assays, myxinidin2 showed no toxicity at concentrations up to 50 μM, while myxinidin3 showed 17% hemolysis at 50 μM, which is eight times the concentration necessary for antibiotic activity (Figure 1A, 1B).

Figure 1C shows the killing kinetics of myxinidin2 and myxinidin3 at 1× and 2× MIC against *S. aureus* CCARM 3018, *A. baumannii* 719705, and *P. aeruginosa* 4076. Both peptides exhibited rapid-killing kinetics against gram-negative bacteria, but somewhat slower killing kinetics against *S. aureus* CCARM 3018. At 2× MIC, the peptides showed antibacterial activity at 10 min and bactericidal activity at 20 min against the three strains.

### Anti-biofilm effect of the peptides against antibiotic-resistant bacteria

**Figure 2:**
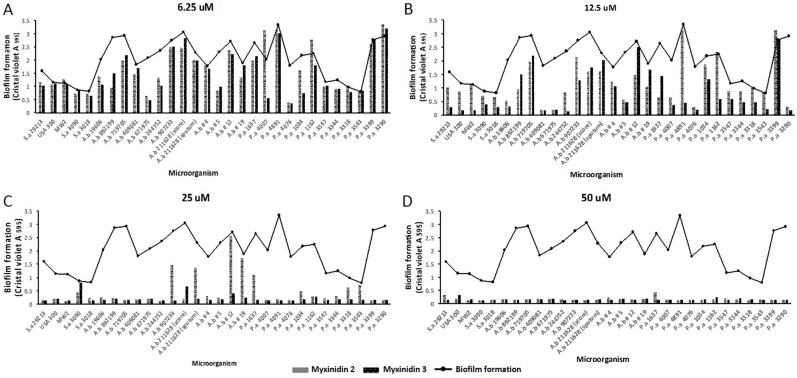
Anti-biofilm activity of myxinidin2 and myxinidin3 Inhibition of biofilm formation was examined at the indicated concentrations of the peptides **(A)** 6.25 μM, **(B)** 12.5 μM, **(C)** 25 μM, **(D)** 50 μM. Each experiment was performed in triplicate, with at least three independent experiments.

We next explored the anti-biofilm activities of myxinidin2 and myxinidin3. Nearly all the tested bacterial strains formed robust biofilms; in particular, the biofilm mass of the antibiotic-resistant strains all had an optical density greater than 1 (Supplementary Figure 2). When the biofilms of the tested strains were treated with the peptides at concentrations ranging from 6.25 μM to 50 μM, an anti-biofilm effect was observed even at the lowest concentration, and the biofilm formation was decreased at 12.5 μM. At 25 μM, the biofilm was dramatically reduced, and at 50 μM, formation was completely blocked. At 12.5 μM, the peptides showed anti-biofilm activity against *P. aeruginosa* 4076. At 25 μM, the peptides were remarkably effective against *S. aureus* CCARM 3018 and *A. baumannii* 719705 (Figure 2A-2D).

### The antimicrobial activity of myxinidin2 and myxinidin3 is sustained in the presence of high salt concentrations

NaCl is generated by inflammation within infected skin wounds [19]. Myxinidin2 and myxinidin3 were incubated with selected concentrations of NaCl in sodium phosphate buffer to test whether they retained their antimicrobial activity under high-salt conditions. Both the edema and swelling in the bacterial-infected skin wounds were sustained at high Na^+^ concentrations. Myxinidin2 and myxinidin3 showed antimicrobial activity against *S. aureus* CCARM 3018 at 75 mM NaCl and against *A. baumannii* 719705 and *P. aeruginosa* 4076 at 150 mM NaCl (Supplementary Figure 3). These peptides thus retain strong antibacterial activity in the presence of high NaCl concentrations and in phosphate-buffered saline, which is similar to the environment within the body.

### Antimicrobial activity of myxinidin2 and myxinidin3 in gelatin used for wound healing

**Figure 3:**
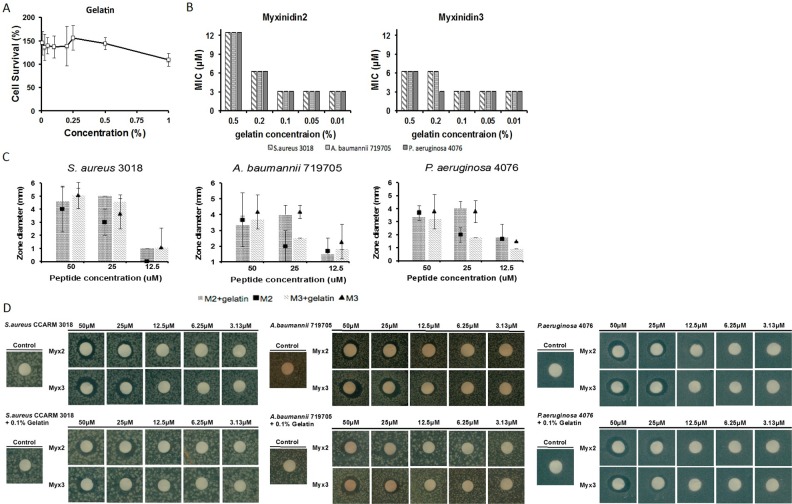
Antimicrobial activity of myxinidin2 and myxinidin3 in gelatin **(A)** Cytotoxicity of 0% to 1% gelatin against normal human keratinocytes. **(B)** Sustained antimicrobial effect at each gelatin concentration was verified by determining the MIC. **(C)** and **(D)** Disc assays showing the antibacterial effects of myxinidin2 and myxinidin3 in 0.1% gelatin. Each experiment was performed in triplicate, with at least three independent experiments.

Gelatin is used as a humectant when treating skin wounds [20]. One of the functions of skin is to retain moisture to maintain the water balance in the body and to boost immune function. Ideally, wound wetting agents should suppress bacterial infection and should be permeable to water vapor. In addition, wound wetting agents must be biodegradable, with no antigenicity or toxicity, and facilitate healing [21]. As shown in Figure 3A, gelatin, at 0.0078%-1%, was not toxic to NHKs. In addition, myxinidin2 and myxinidin3 retained antimicrobial activity in 0.1% gelatin (Figure 3B). The antimicrobial activity of the peptides in agarose plates containing 0.1% gelatin was measured using the disk diffusion method (Figure 3C, 3D) [22]. In gelatin, both peptides showed antimicrobial activity against *S. aureus* CCARM 3018 that was similar to or higher than in broth. At 50 μM, myxinidin2 retained antimicrobial activity against *A. baumannii* 719705 and *P. aeruginosa* 4076 in gelatin, showed increased activity at 25 μM, and similar effects at 12.5 μM. The activity of myxinidin3 against *A. baumannii* 719705 and *P. aeruginosa* 4076 in gelatin was slightly lower than was observed in broth. Nonetheless, myxinidin3 continued to show antibiotic activity against these drug-resistant strains.

### AMPs stimulate NHK migration and wound re-epithelialization through EGFR activation

**Figure 4:**
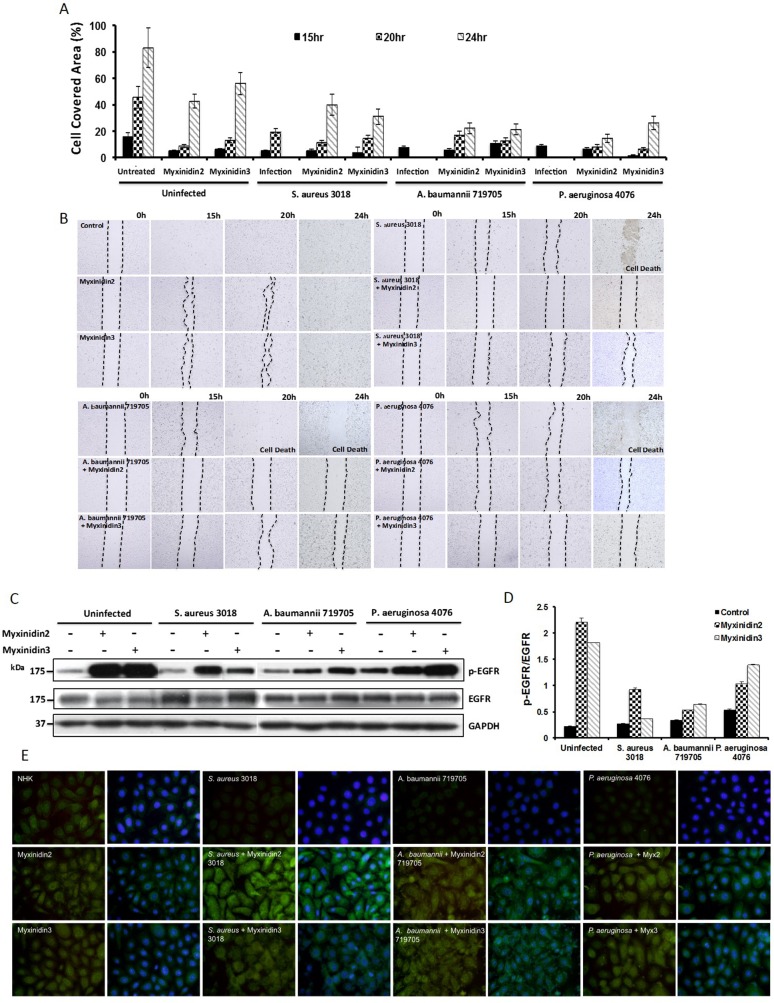
Effects of myxinidin2 and myxinidin3 on migration of infected keratinocytes **(A)** Graph showing the effects of the indicated peptides on wound area coverage by infected keratinocytes at the indicated times. **(B)** Visualization of wound closing through keratinocyte migration at the indicated times after wounding. Dotted lines denote the wound. Cells were infected with the indicated bacterial with or without myxinidin2 and myxinidin3 treatment. **(C)** and **(D)** Western blot analysis showing that myxinidin2 and myxinidin3 stimulated EGFR phosphorylation. **(E)** Immunofluorescent staining revealed activation of EGFR by myxinidin2 and myxinidin3 at the surface of infected keratinocytes. EGFR was detected using anti-EGFR antibody (green). The nuclei were stained with Hoechst (blue). Each experiment was performed in triplicate, with at least three independent experiments.

To investigate the effects of myxinidin2 and myxinidin3 on cell proliferation, monolayers of plated primary NHKs were scratched and pre-treated with *S. aureus* CCARM 3018, *A. baumannii* 719705, or *P. aeruginosa* 4076 [23]. As shown in Figure 4A and 4B, bacteria-infected cells did not re-epithelialize after being scratched and eventually died. By contrast, infected cells treated with peptides began to proliferate within 24 h, without further damage. For *S. aureus-* and *A. baumannii*-infected cells, the cell migration induced by myxinidin3 was higher than was induced by myxinidin2, whereas a stronger recovery by *P. aeruginosa*-infected cells was induced by myxinidin2. These observations suggest that the re-epithelialization promoted by myxinidin2 and myxinidin3 reflects their ability to stimulate primary keratinocyte migration rather than keratinocyte regeneration. Consistent with the observation that cell migration was increased in scratch wounds treated with myxinidin2 and myxinidin3, these peptides were able to inhibit the growth of bacteria in NHKs. In infected cells, the number of bacteria rapidly increased during the period 2 h to 4 h after infection, which fatally damaged the keratinocytes. However, in infected cells treated with myxinidin2 or myxinidin3, the keratinocytes appeared normal and were able to migrate (Supplementary Figure 4).

Although the total EGFR levels were equal in all samples, the levels of p-EGFR were higher in NHKs treated with myxinidin2 or myxinidin3. Within infected cells, there was a sharp decline in p-EGFR levels. In *S. aureus* (gram-positive)-infected cells, myxinidin2 stimulated higher p-EGFR levels than did myxinidin3. However, in *A. baumannii* and *P. aeruginosa* (gram-negative)-infected cells, myxinidin3-treated cells expressed more p-EGFR than did myxinidin2-treated cells (Figure 4C, 4D). Fluorescent staining revealed the presence of EGFR on the surface of uninfected NHKs, but little or no staining was seen in the infected cells (Figure 4E). Apparently, myxinidin2 or myxinidin3 act through EGFRs to aid wound healing and cell reproduction within infected wounds.

### Myxinidin2 and myxinidin3 ameliorate cutaneous infection with antibiotic-resistant bacteria *in vivo*

**Figure 5:**
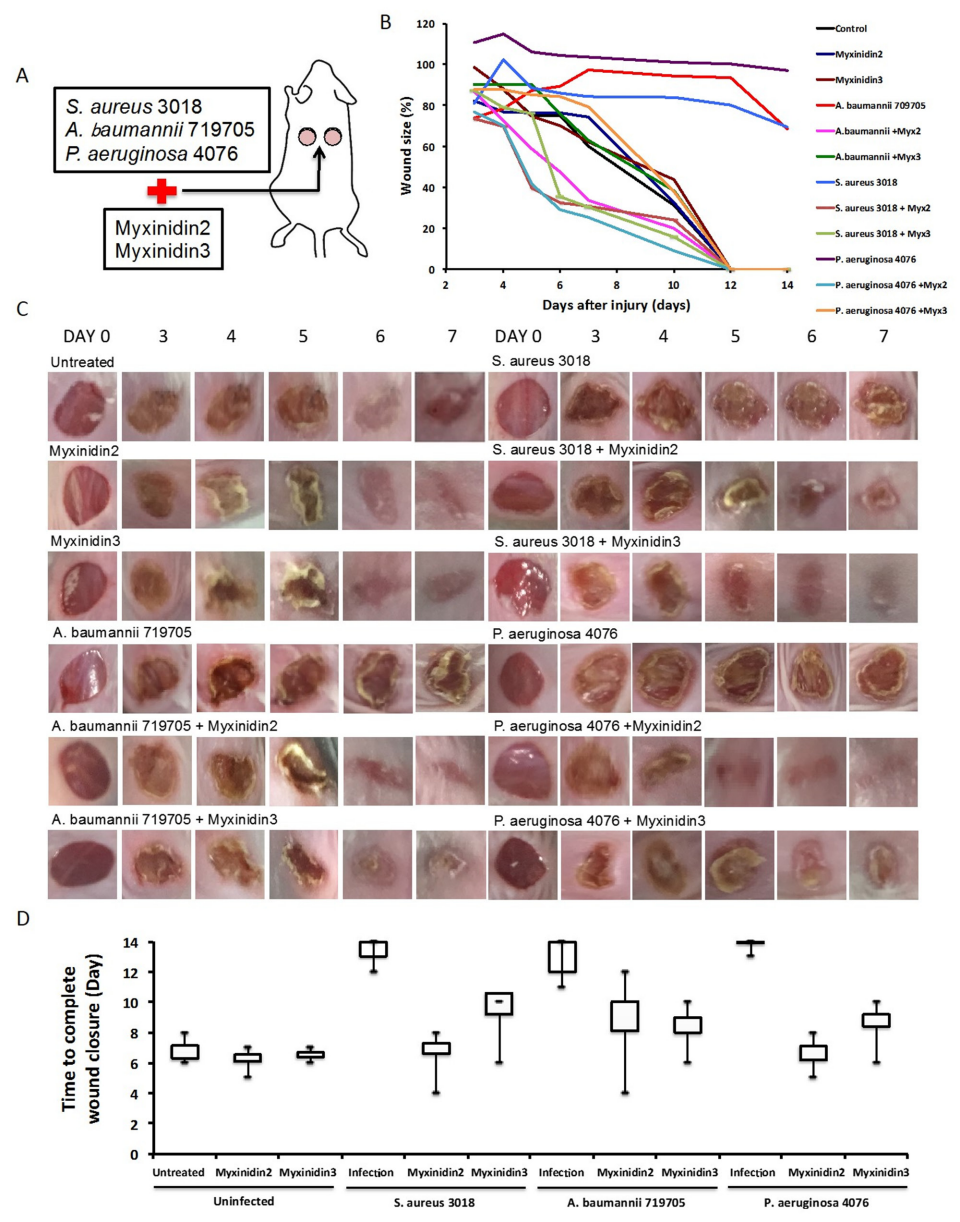
Effect of myxinidin2 and myxinidin3 on wound healing *in vivo* **(A)** Schematic illustration of wound healing model. **(B)** Percent change in wound size over time. **(C)** Images of wounds 0, 3, 4, 5, 6, 7 days after injury. **(D)** Time to complete wound closure in each group. Values shown are the means of three individual experiments.

We next tested the antibacterial activity of myxinidin2 and myxinidin3 against *S. aureus* CCARM 3018, *A. baumannii* 719705, and *P. aeruginosa* 4076 infections in skin wounds on mice (Figure 5A). Without AMP treatment, the size of the wounds infected with the antibiotic-resistant strains did not decrease over a period of 2 weeks, by which time the wounds had become severely inflamed. By contrast, for all three strains, the appearance of the infected wounds treated with myxinidin2 or myxinidin3 was similar to that of the uninfected control wounds (Figure 5B, 5C). Myxinidin2-treated wounds closed within 4-5 days and were completely healed within 12 days, while myxinidin3-treated wounds began to close within 6 days, and the wounds were completely healed in 11 days (Figure 5D).

**Figure 6:**
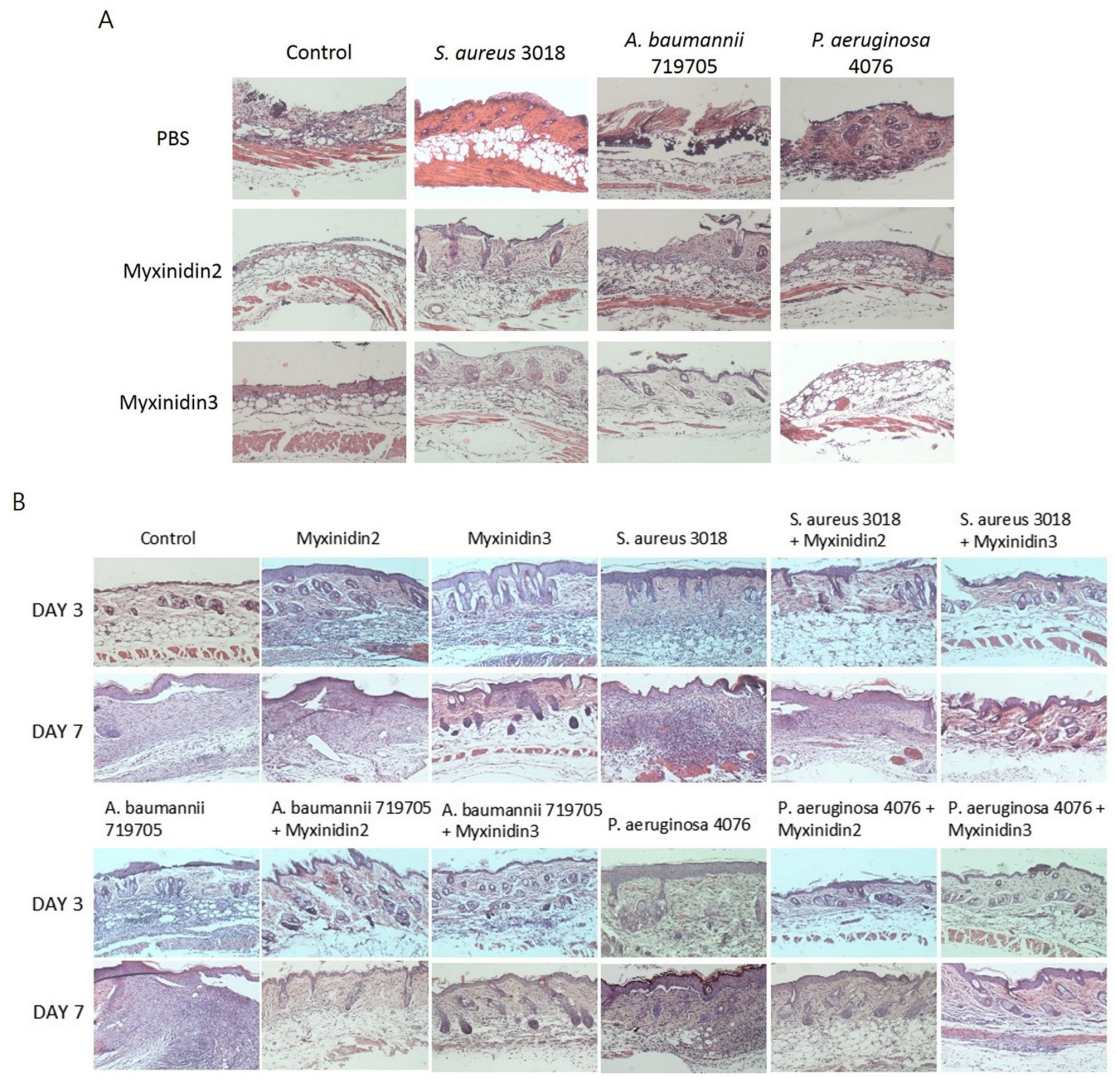
Histological evaluation of infected wound sections **(A)** H&E stained sections of infected wounds, with and without myxinidin2/3 treatment. **(B)** H&E stained section of infected wounds 3 and 7 days after injury, with and without myxinidin2/3 treatment.

The epidermis of uninfected and infected wounded skin is shown in Figure 6A. The skin of infected mice showed ecdysis or necrosis and exhibited thinner dermis and subcutaneous adipose tissue than the skin of control mice. When stained with H&E on days 3 and 7, both myxinidin2- and myxinidin3-treated mice showed a thick skin layer and were protected from extensive tissue necrosis (Figure 6B) [24].

### Inhibitory effects of pro-inflammatory cytokines and chemokines induced by antibiotic-resistant bacteria

**Figure 7:**
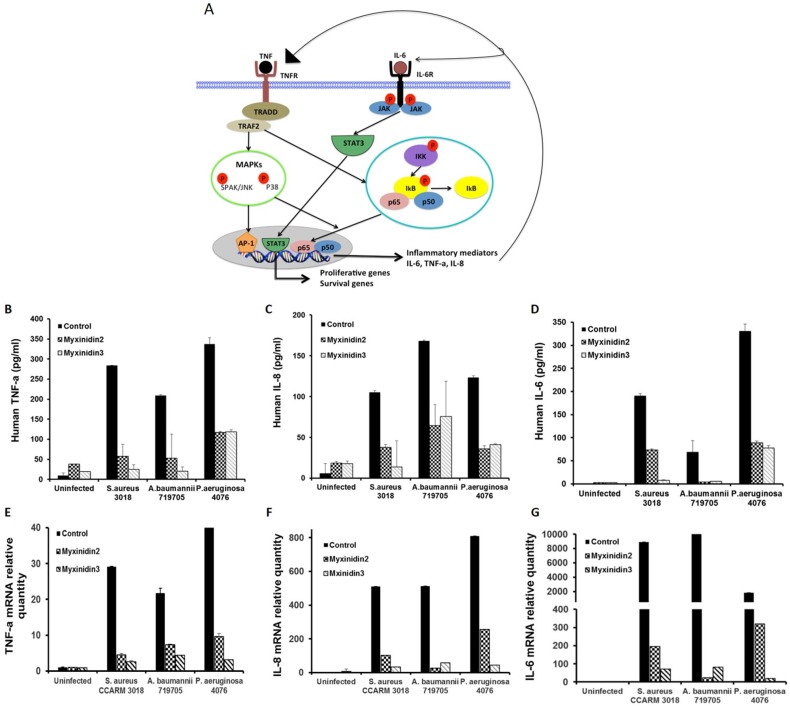
Model of inflammatory signal pathways in keratinocytes **(A)** Normal human keratinocytes were infected with *S. aureus* CCARM 3018, *A. baumannii* 79705 or *P. aeruginosa* 4076 and then treated with myxinidin2/3. Expression levels of cytokines and chemokines were quantified using ELISAs. **(B)** TNF-α, **(C)** IL-8, **(D)** IL-6. Levels of cytokine and chemokine mRNAs were quantified using qRT-PCR. **(E)** TNF-α, **(F)** IL-8, **(G)** IL-6. Each experiment was performed in triplicate, with at least three independent experiments.

The mRNA and protein expression levels of inflammatory mediators such as IL-6, IL-8, and TNF-α are reportedly modulated during both cutaneous infection and wound healing (Figure 7A) [25]. Following infection of NHKs by *S. aureus*, *A. baumannii*, or *P. aeruginosa*, expression of IL-6, IL-8, and TNF-α was significantly upregulated, and this response was inhibited by myxinidin2 and myxinidin3. By contrast, the peptides had no effect on basal IL-6, IL-8, and TNF-α expression in uninfected NHKs. These results indicate that myxinidin2 and myxinidin3 inhibit expression of inflammatory cytokines and chemokines induced in NHKs by infection with drug-resistant bacteria (Figure 7B-7G).

### Myxinidin2 and myxinidin3 promote anti-inflammatory activity via STAT3, p38, JNK/SAPK, and NF-κB

**Figure 8:**
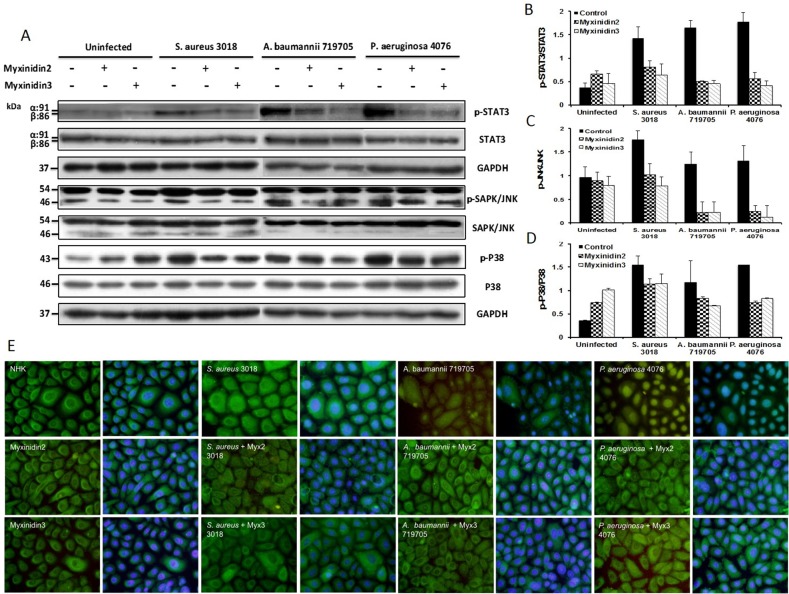
**(A)** Western blot analysis of the effects of myxinidin2 and myxinidin3 on levels of phosphorylated STAT3 and MAPK (SAPK/JNK and P38). Cells were pre-infected with the indicated bacteria before addition of the peptides. The results are configured as bar graphs in **(B)**, **(C)** and **(D)**. **(E)** Myxinidin2 and myxinidin3 inhibit bacteria-induced NF-kB translocation in normal human keratinocytes. NF-kB was detected using a polyclonal anti-NF-kB p65 antibody. Its intracellular localization (green) was compared with Hoechst stained nuclei (blue). Each experiment was performed in triplicate, with at least three independent experiments.

We also examined the expression of intrinsic anti-inflammatory proteins. Bacterial infection increased the expression of STAT3, JNK/SAPK, p38, and NF-κB in primary NHKs. Our results showed that the protein expression of these inflammatory mediators was significantly higher (approximately 1.5-fold) in infected than uninfected cells. Myxinidin2 and myxinidin3 inhibited the inflammatory response to infection with *S. aureus* CCARM 3018, *A. baumannii* 719705, and *P. aeruginosa* 4076 (4 × 10^7^ colony-forming units (CFU)/mL) by suppressing the phosphorylation (activation) of STAT3, JNK, and p38 (Figure 8A-8D). In addition, in uninfected NHKs, NF-κB staining was primarily observed in the cytoplasm. Following infection, however, nuclear translocation of NF-κB was rapidly induced. Co-incubation of NHKs with bacteria and myxinidin2 or myxinidin3 effectively blocked the bacteria-induced NF-κB nuclear translocation (Figure 8E).

## DISCUSSION

Proper healing of infected wounds involves precise interactions among bactericidal activity, inflammation, epithelialization, and tissue granulation and remodeling. Methicillin-resistant *S. aureus* (MRSA), antibiotic-resistant *A. baumannii* [26] and *P. aeruginosa*, and other multidrug-resistant bacteria are causing serious problems worldwide as bacteria gradually acquire new mechanisms of resistance. And when these bacteria are able to develop into a complex biofilm structures, it becomes especially difficult for host defenses and conventional antibiotics to combat them. Consequently, patients with skin infections from one of these strains experience prolonged and potentially lethal illnesses. The present investigation demonstrated that myxinidin2 and myxinidin3 exert strong antibacterial effects against skin infections caused by MRSA and antibiotic-resistant *A. baumannii* and *P. aeruginosa*. In addition to their antibiotic effects, these short peptides also possess potent wound-closure activity both *in vitro* and *in vivo*. These peptides did not affect the viability of NHKs or RBCs at concentrations of up to 50 μM (Figure 1A, 1B). And because they act on the bacterial membranes, their beneficial effects develop rapidly (within 10 min) (Figure 1C) [27].

The ability of resistant bacteria to form biofilms on the surfaces to which they adhere disrupts the function of conventional antibiotics. Biofilm formation by *S. aureus*, *A. baumannii*, and *P. aeruginosa* on plastic was inhibited by myxinidin2 and myxinidin3 at concentrations of 6.25-50 μM (Figure 2A-2D). In addition, skin edema associated with infections is characterized by water retention and swelling that creates a microenvironment with a high salt concentration. Therefore, to be useful for treating such infections, synthetic peptides must retain their effectiveness in the presence of high salt concentrations. We assessed the antimicrobial activity of myxinidin2 and myxinidin3 in infected cell monolayer “wounds” at various salt concentrations (Supplementary Figure 3). We also showed that myxinidin2 and myxinidin3 inhibited the growth of microorganisms in gelatin and accelerated wound healing. Gelatin is largely composed of glycine and proline, which are needed not only for proper skin, hair, and nail growth, but also for optimal immune function as a humectant. Glycine, which makes up approximately 1/3 of the amino acids in gelatin, has anti-inflammatory effects, and evidence suggests it can accelerate wound healing (Figure 3) [21].

The healing of skin wounds proceeds through four stages: blood clotting (hemostasis), inflammation, tissue growth (proliferation), and tissue remodeling (maturation). We demonstrated that *S. aureus*, *A. baumannii*, and *P. aeruginosa* activate the STAT3, MAPK, and NF-κB signaling pathways, which promote the expression and secretion of pro-inflammatory cytokines in keratinocytes [28–30]. Inflammatory cytokines and chemokines, including IL-6, IL-8, and TNF-α, play major roles in the development of chronic skin inflammation [31]. IL-6, which is secreted from keratinocytes, is present on the cell surface and within intracellular compartments and induces intracellular signaling cascades that give rise to inflammatory cytokine production. Our findings in the present study are consistent with earlier observations that in healthy keratinocytes, IL-6 is only minimally expressed, but at sites of infection, IL-6 overexpression is stimulated by various other cytokines, including IL-1, TNF-α, and INF-γ [32]. IL-8 is a strong chemotactic factor for neutrophils, basophils, and lymphocytes and plays a key role as an anti-inflammatory factor against pathogens in the skin. We also demonstrated that infection-induced IL-8 overexpression was reduced by treatment with the myxinidin2 or myxinidin3. TNF-α is the major pro-inflammatory cytokine in psoriasis; it acts to upregulate other inflammatory mediators and regulates immune responses by stimulating Th1 lymphocytes to release various inflammatory and immune mediators [33]. Both myxinidin 2 and myxinidin3 effectively suppressed TNF-α expression in all infected cells. In that way, these peptides mitigated the inflammatory response, as evidenced by the reduced secretion of IL-6, IL-8, and TNF-α.

Thus, myxinidin2 and myxinidin3 modulated inflammatory signaling via IL-6, IL-8, TNF-α, STAT3 [34], p38 [35], JNK, and NF-κB and enhanced wound-closure activity by inducing the migration of primary keratinocytes [36], which involves transactivation of EGFR [37]. STAT3 is a multifunctional protein upregulated in inflammation, and its downregulation leads to anti-inflammatory activity [38] and cell growth through stimulation of EGF [39, 40]. Infected keratinocytes treated with myxinidin2 or myxinidin3 showed higher levels of STAT3 phosphorylation than uninfected cells.

We also showed the effects of myxinidin2 and myxinidin3 on phosphorylation of p38 and JNK in the MAPK signaling pathway, which controls cytokine production and secretion during the inflammatory responses. Activation of MAPKs stimulates transcription factors in the cytoplasm and nucleus to induce the expression of target genes related to innate immunological responses. Infections caused by antibiotic-resistant bacteria induced p38 and JNK activation, but myxinidin2 and myxinidin3 suppressed phosphorylation of p38 and JNK, thereby suppressing production of IL-6, IL-8, and TNF-α [41]. The major regulatory transcription factor NF-κB modulates the expression of numerous cytokines, chemokines, adhesion molecules, and granulopoiesis factors, which support host immune function against bacteria [42, 43]. Our findings showed that myxinidin2 and myxinidin3 prevented NF-κB nuclear translocation otherwise induced by pathogen infection. The identification of myxinidin2 and myxinidin3 as mediators that suppress inflammatory responses in wounded skin provides a better understanding of the wound healing process.

We found that myxinidin2 and myxinidin3 promote the re-epithelialization of surrogate scratch wounds by primary NHKs *in vitro*. Infected cells did not exhibit cell migration; however, infected cells treated with the peptides, especially myxinidin3, exhibited robust cell migration. To migrate and proliferate, keratinocytes require activation of EGFR [44]. Keratinocytes infected with drug-resistant bacteria had higher levels of p-EGFR than healthy keratinocytes, which halts cell reproduction and growth. In addition, infected cells treated with myxinidin2 or myxinidin3 showed greater expression of EGFR than infected cells [45].

To study the effects of myxinidin2 and myxinidin3 on infected wound healing in a mouse model, we created wounds using a biopsy punch, which we then infected with multidrug-resistant bacteria [46]. In this mouse skin wound model, both myxinidin2 and myxinidin3 inhibited the resistant bacteria and enhanced wound healing [47]. Moreover, histological examination showed that these peptides prevented skin necrosis and reduced inflammatory cell infiltration seen 12 days post-infection. The peptide-treated wounds showed significantly better epithelization than the untreated infected skin tissues. Given the potential applications for treatment of systemic infections, myxinidin2 and myxinidin3 may be of great use in the future.

## MATERIALS AND METHODS

### Materials

Gelatin from bovine skin and 3(4,5-dimethylthiazol-2-yl)-2,5-diphenyltetrazolium bromide (MTT) were purchased from Sigma-Aldrich (St. Louis, MO, USA). Hoechst 33342 was from Molecular Probes by Life Technologies (Waltham, MA, USA). IL-6, IL-8, and TNF-α ELISA kits were from R&D systems, Inc., (Minneapolis, MN, USA). Rabbit anti-phosphorylated STAT3 (p-STAT3) polyclonal antibody (cat. no. 4113) was from Cell Signaling Technology, Inc., (Danvers, MA, USA).

### Ethics statement

This study conformed to the ethical standards of the Institutional Ethics Committee of Chosun University, and the protocol was approved by the Institutional Ethics Committee of Chosun University. All mouse experiments were carried out in strict accordance with the National Institutes of Health Guidelines for the Ethical Treatment of Animals and the guidelines of the Center for Experimental Animals of Chosun University for Medical Science (Permit Number: CIACUC2016-A0011).

### Preparation of peptides

The synthetic AMPs myxinidin2 and myxinidin3 were synthesized as described previously [16]. After filtration through a 0.22-μm pore filter, myxinidin2 (KIKWILKYWKWS-NH2) and myxinidin3 (RIRWILRY WRWS-NH2) were prepared at a concentration of 1 mM in sterilized distilled water. These stock solutions were diluted in buffer, as needed.

### Bacterial strains and culture conditions

The *A. baumannii* strains were obtained from patients at EulJi University hospital. The *P. aeruginosa* and methicillin-resistant *S. aureus* (MRSA) strains were obtained from the university hospital. Bacteria were grown overnight in Mueller Hinton (MH) medium at 37°C while shaking. Mid-log phase bacterial cells were washed three times with phosphate-buffered saline (PBS) and resuspended in medium or buffer at 1 × 10^8^ CFU/mL.

### Antimicrobial and bactericidal activity

For these experiments, drug-resistant strains were cultured in the appropriate medium at 37°C. The minimum inhibitory concentrations (MICs) and minimum bactericidal concentrations (MBCs) of the peptides against the tested microorganisms were determined using the methods of the National Committee of Clinical and Laboratory Standards (NCCLS). AMPs were dissolved in MHB medium and serially diluted in the medium before inoculation. The concentrations of the peptides ranged from 0.39 μM to 50 μM. The MBCs were verified on an MH agar plate.

### Anti-biofilm activity

Biofilm formation was quantitatively analyzed as described previously [17]. Briefly, aliquots (90 μL) of bacterial cell suspension (1×10^6^ CFU/mL) in MHB supplemented with 0.2% glucose were added to a 96-well flat-bottomed tissue culture plate. The peptides were serially diluted (0.39-50 μM) and added to the wells, after which the plates were incubated for 24 h at 37°C to allow biofilm formation. The biofilms were then fixed in 100% methanol for 15 min, washed three times with distilled water, and stained with 0.1% crystal violet + 0.25% acetic acid for 2 h. To assess biofilm formation, the stained biofilms were dissolved in 100% ethanol, and the absorbance at 570 nm was measured using a Versa-Max microplate ELISA reader (Molecular Devices, Sunnyvale, CA, USA).

### Kinetics assays

Growth kinetics were assayed using *S. aureus* CCARM 3018, *A. baumannii* 719705, and *P. aeruginosa* 4076 in the presence of selected AMPs at 1× MIC and 2× MIC. The bacterial cells were cultured overnight until they reached the exponential growth phase, and then incubated for various time periods (0, 10, 20, 30, 40, 50, 60, 90, and 120 min) at 37°C. The total bacterial population was then plated on MHB agar plates and incubated overnight, and the colonies were counted.

### Culture of NHKs

Primary normal human keratinocytes (NHKs) were cultured in Epilife medium containing 60 μM calcium supplemented with the growth factors (Gibco) used in most assays. Cells were maintained in a humidified chamber at 37°C and 5% CO_2_.

### Hemolysis and cytotoxicity assays

The hemolytic activity of the AMPs was assessed using heparinized red blood cells (RBCs) from mice. The RBCs were centrifuged (×2000 *g*) for 10 min and washed three times with PBS until the supernatant was clear. The AMPs were dissolved in PBS, serially diluted, and added to each well along with 8% blood. After incubation with smooth agitation for 1 h at 37°C, the sample was centrifuged at 1500 rpm for 10 min. The supernatant was transferred to a 96-well plate, and the absorbance at 414 nm was measured.

The cytotoxic activity of the peptides toward immortalized keratinocytes was examined using a colorimetric assay with 0.5 mg/mL MTT dye. Briefly, HaCaT cells (1×10^5^ cells/mL) were seeded into a 96-well plate and incubated at 37°C in 5% CO_2_ until confluent. AMPs were then added to the wells to concentrations of 0 to 50 μM for 24 h, after which MTT dye was added to each well and incubated for 4 h at 37°C. The formazan crystals formed were dissolved with dimethyl sulfoxide (DMSO), and the absorbance at 570 nm was measured.

### Sodium chloride sensitivity of the AMPs

The antimicrobial activities of each peptide against *S. aureus* CCARM 3018, *A. baumannii* 719705, and *P. aeruginosa* 4076 in the presence of the indicated NaCl concentrations were investigated using the NCCLS method. MIC assays were conducted in sodium phosphate buffer and PBS, in the absence or presence of 25, 50, 75, 100, or 150 mM NaCl. Mid-log phase cultures in MH broth were diluted with medium to 2×10^5^ CFU/mL, after which the peptides were added to concentrations up to 50 μM, and the cells were incubated for 18-24 h at 37°C. Sodium chloride sensitivity was defined as the lowest peptide concentration that entirely inhibited bacterial growth.

### Anti-bactericidal activity in gelatin

Overnight cultures of *S. aureus* CCARM 3018, *A. baumannii* 719705, and *P. aeruginosa* 4076 cells grown at 37°C in MH broth medium were washed with PBS and resuspended in 10 mL of MHB medium at 4 × 10^5^ CFU/mL. Then, 10 mL of the prepared bacterial cells and 10 mL of 0.2% agarose were mixed and plated. To evaluate bactericidal activity, 6-mm round disks were permeated with selected concentrations of the AMPs. The disks were placed on the agarose plate and incubated at 37°C overnight. The bactericidal activity was assessed by measuring the diameter of the clear zone surrounding the paper disks.

### Measurement of IL-6, IL-8, and TNF-α secretion

IL-6, IL-8, and TNF-α levels were measured using human IL-6, IL-8, and TNF-α immunoassay kits (R&D systems, Minneapolis, MN, USA) according to the manufacturer's instructions. Samples of supernatants were taken from cultures of *S. aureus* 3018, *A. baumannii* 719705, and *P. aeruginosa* 4076 after incubation with AMPs at 2× MIC for 3 h or 6 h.

### Assessment of STAT3, JNK, p38, and EGFR activation using western blot analysis

After lysis of NHKs in cell/tissue lysis buffer and centrifugation at 13,000 rpm for 15 min at 4°C, the protein concentrations in the supernatants were determined using Bradford assays. The proteins (30-40 μg) were then separated by 10% or 12% SDS-polyacrylamide gel electrophoresis and transferred onto PVDF membranes (100 V for 2 h). The membranes were first blocked with 5% skim milk in Tris-buffered saline containing Tween-20 (TBST) at room temperature for 1 h. They were then incubated with the following primary antibodies overnight at 4°C: mouse anti-p-STAT3 monoclonal antibody (1:500 dilution), rabbit anti-STAT3 polyclonal antibody (1:1000 dilution), rabbit anti-phosphorylated stress-activated protein kinase/JNK (p-SAPK/JNK) polyclonal antibody (1:1000 dilution), rabbit anti-SAPK/JNK polyclonal antibody (1:1000 dilution), rabbit anti-phosphorylated p38 MAP kinase (phospho-p38) polyclonal antibody (1:1000 dilution), rabbit anti-p38 MAP kinase (p38) polyclonal antibody (1:1000 dilution), rabbit anti-phosphorylated EGFR (p-EGFR) monoclonal antibody (1:1000 dilution), rabbit anti-EGFR monoclonal antibody (1:1000 dilution), and rabbit anti-glyceraldehyde-3-phosphate dehydrogenase (GAPDH) polyclonal antibody (1:1000 dilution). After washing five times with TBST, the PVDF membrane was incubated with the secondary antibody (1:5000 dilution) for 2 h at room temperature. After again washing five times with TBST, ECL solution was added. To detect the signal, working in a darkroom, X-ray film was exposed to the membrane and enclosed in a film cassette.

### RNA isolation and quantitative real-time (qRT)-PCR

Total RNA (1 μg) was isolated using a TRIzol RNA extraction kit (Life Technologies, Waltham, MA, USA) according to the manufacturer's protocol, after which first-strand cDNA was synthesized using a Topscript™ cDNA synthesis kit (Enzynomics, Daejeon, Korea). Each qRT-PCR was carried out in a total volume of 20 μL containing TOPreal™ qPCR 2× premix (SYBR Green), cDNA in RNase-free water, and 1 μL of each forward and reverse primer. All qRT-PCR experiments were performed in a 7500 RT-PCR system (Applied Biosystems, Thermo Fisher Scientific, Inc., Waltham, MA, USA). Each sample was analyzed in triplicate. The cycling protocol entailed: 10 min at 95°C followed by 40 cycles of 95°C for 10 s, 60°C for 30 s, and 72°C for 30 s. Melting curves were generated using 71 PCR cycles, with the temperature increasing in 0.5°C increments (60-95°C). For quantification of mRNA, transcript levels were normalized to the level of GAPDH mRNA.

### *In vitro* cell migration assay

NHKs (1×10^6^ cell/mL) suspended in keratinocyte medium containing Human Keratinocyte Growth Supplement (HKGS) were seeded into a 12-well plate and incubated at 37°C in 5% CO_2_ until 95% confluent. A scratch wound was then created on each confluent monolayer using a 200-μL pipette tip, after which the cells were infected with bacteria (1×10^5^ CFU/mL) in fresh medium without HKGS and treated with AMPs at 2× MIC. The wound area was then visualized at the indicated times under an inverted microscope (Olympus), and the percentage of cell-covered area at each time point was determined using Image J.

NHKs (8×10^5^ cell/mL) were seeded into a 96-well plate containing keratinocyte medium without HKGS and incubated in an IncuCyte ZOOM™ cell analysis system (Essen BioScience, USA). A scratch wound was then made using a 96-well wound maker on monolayers at 90-100% confluence. The scratch wounds were infected with bacterial cells (1×10^3^ CFU/mL) and then treated with the AMPs at 2× MIC. Cell migration was observed, and photographed for 24 h.

### Immunofluorescent staining for nuclear translocation of NF-κB and cellular localization of EGFR

Immunofluorescence analysis of NF-κB and EGFR localization was conducted as described previously [48]. NHKs were seeded at 5×10^4^ cells/mL into four-chambered glass slides, and then NF-κB and EGFR were detected 2-4 h after infection in the presence and absence of 1× MIC of myxinidin2 or myxinidin3. Thereafter, the cells were fixed in 100% methanol for 10 min at −20°C and washed three times with PBS. For analysis of NF-κB, the cells were washed in PBS containing 0.5% Triton X-100 to permeabilize the membrane and were then blocked for 1 h at room temperature with 4% BSA in PBS. The blocked cells were incubated with a rabbit polyclonal anti-human NF-κB p65 antibody or rabbit monoclonal anti-human EGFR antibody (1:1000 dilution in PBS with 0.1% BSA) overnight at 4°C. The cells were then incubated with a FITC-conjugated affinity-purified goat anti-rabbit IgG (1:200 dilution in PBS with 0.1% BSA) for 2 h at room temperature in the dark. After washing with PBS, nuclei were stained with Hoechst 33342, which labels nuclear DNA. Finally, the cells were visualized under a microscope (Olympus).

### Wound healing *in vivo*

Six- to seven-week-old female BALB/c mice were used in this study. Full- thickness dermal excisional wounds were made on opposite sides of the midline using a 5-mm punch biopsy instrument. Wounds and the surrounding tissues were then harvested on days 3, 7, and 10. The wounds were photographed at the indicated time points, and wound size was determined using ImageJ.

### Histological preparations

Skin biopsy specimens were collected immediately after the mice were sacrificed and fixed in PBS containing 4% formalin. The formalin-fixed biopsy specimens were embedded in paraffin and sectioned, and the obtained sections were stained with H&E.

### Statistical analyses

All experiments were performed in triplicate in at least three independent experiments. Data shown are the mean ± SD. One-way analysis of variance was used to determine the significance of differences. Values of *P* < 0.05 were considered significant.

## CONCLUSION

Our findings demonstrate that, independent of their antimicrobial and anti-biofilm effects in high salt concentrations and the presence of the humectant gelatin, myxinidin2 and myxinidin3 also affect inflammation and promote the healing of wounds infected with antibiotic-resistant *S. aureus*, *A. baumannii*, or *P. aeruginosa*. *In vivo*, moreover, these peptides mitigate inflammatory responses and stimulate wound healing through elimination of the resistant bacteria from skin wounds. We therefore anticipate that myxinidin2 and myxinidin3 will be effective agents for use in the treatment of human skin wounds infected with antibiotic-resistant bacteria.

## SUPPLEMENTARY FIGURES


