# Supplementary material for: Myxinidin2 and myxinidin3 suppress inflammatory responses through STAT3 and MAPKs to promote wound healing
Source: Oncotarget. 2017 Sep 15;8(50):87582–97. doi: 10.18632/oncotarget.20908 (PMC5675655; doi:10.18632/oncotarget.20908)
Supplement: Supplementary file 1 [file oncotarget-08-87582-s001.pdf]

Myxinidin2 and myxinidin3 suppress inflammatory responses through STAT3 and MAPKs to promote wound healing

SUPPLEMENTARY MATERIALS

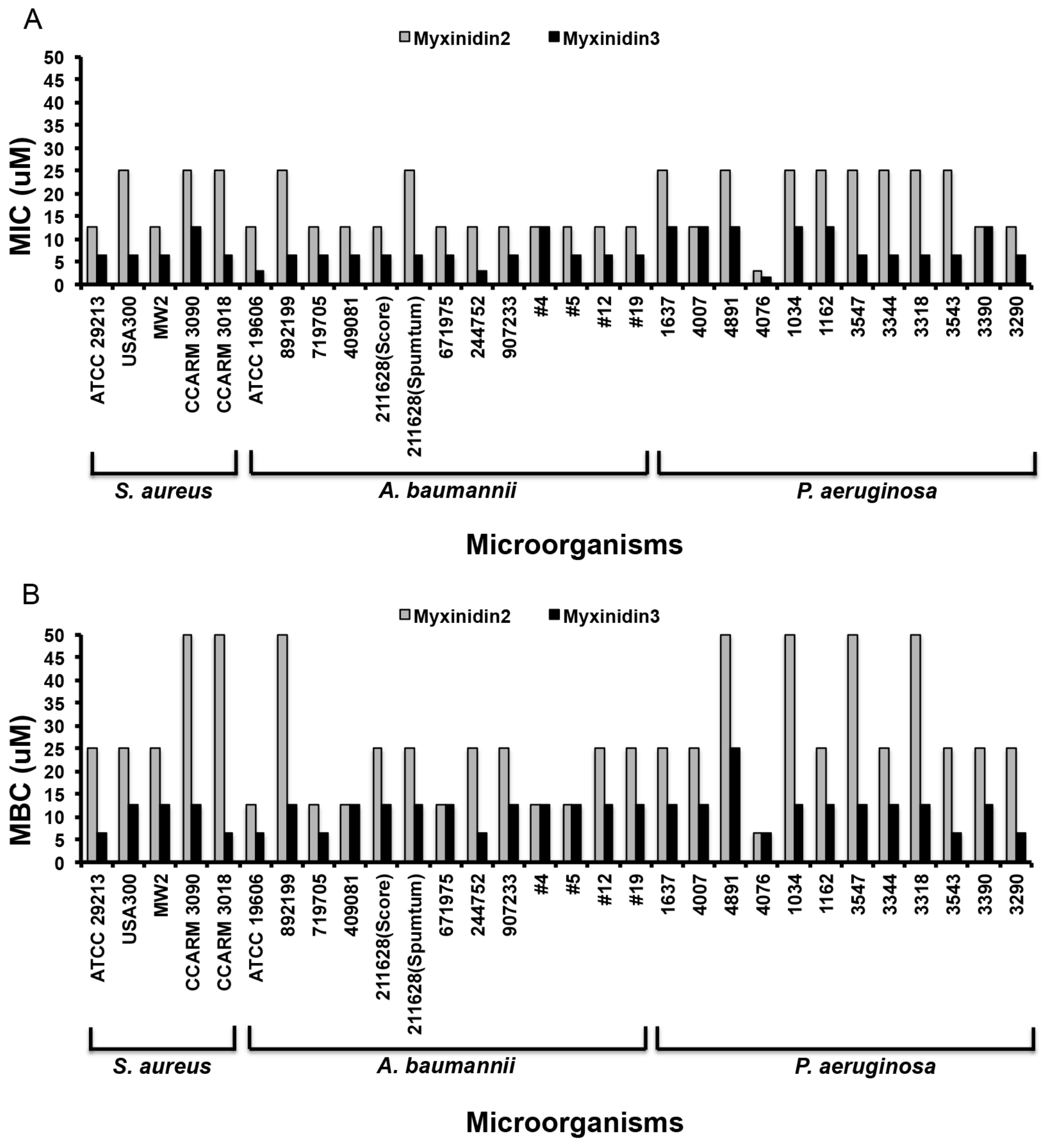

Supplementary Figure 1: Minimum inhibitory concentration (MIC) **(A)** and Minimum bactericidal concentration (MBC) **(B)** of synthetic peptide analogs from Myxinidin isolated from hagfish against drug-resistant bacteria strains**.** *S. aureus*: *Staphylococcus aureus*; *A. baumannii*: *Acinetobactor baumannii*; *P. aeruginosa*: *Pseudomonas aeruginosa*.

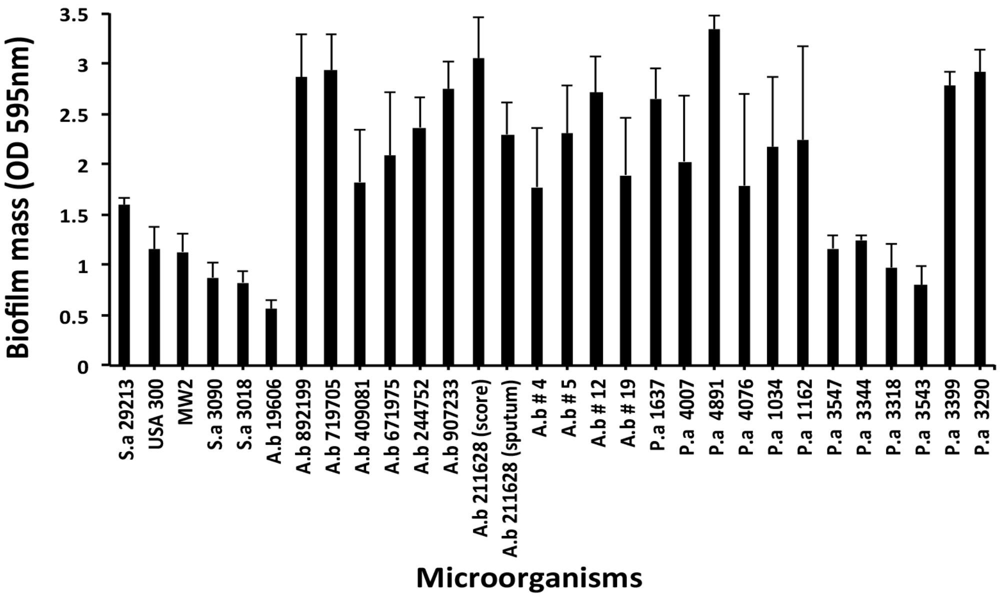

Supplementary Figure 2: The degree of biofilm formation in MHB media with 0.2% glucose by resistant bacteria strains.

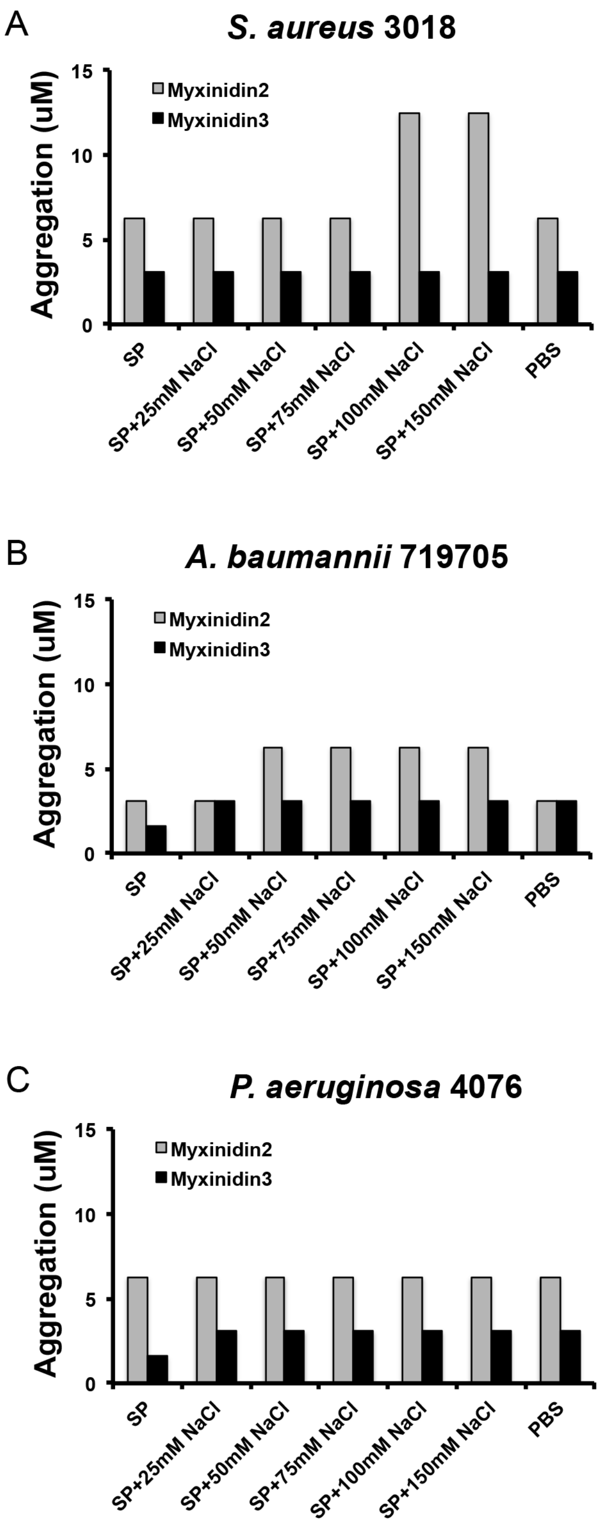

Supplementary Figure 3: Antibacteial activity the peptides on various salt concentration and buffer against **(A)** *S. aureus* CCARM 3018, **(B)** *A. baumannii* 719705, and **(C)** *P. aeruginosa* 4076.

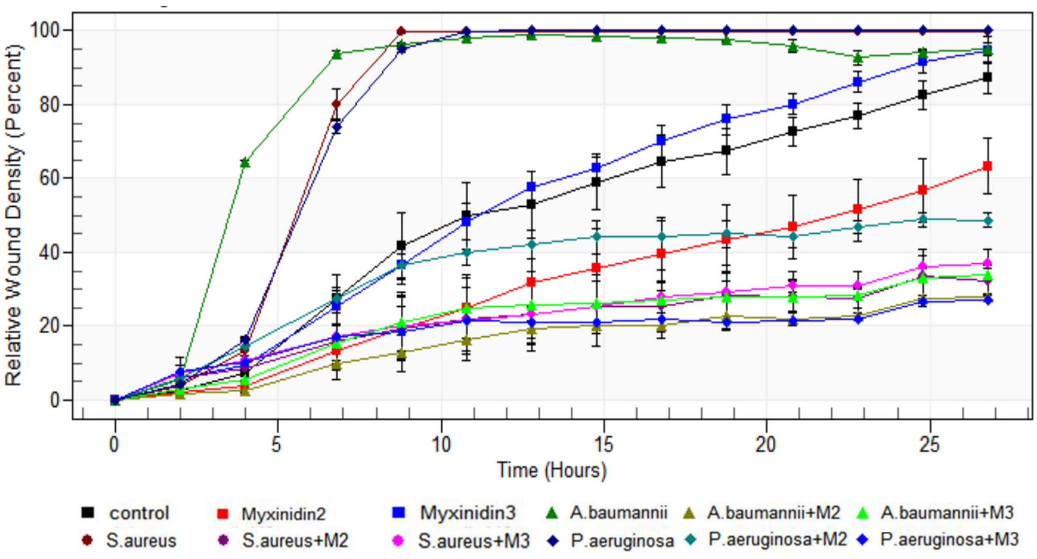

Supplementary Figure 4: To determine the effects of Myxinidin2 and Myxinidin3 on cell migration, a wound healing assay was performed on normal human keratinocyte cells including *S. aureus* CCARM 3018, *A. baumannii* 719705, and *P. aeruginosa* 4076. Representative graph means relative wound density (%) in keratinocyte cells from 0hr to 24hr. human keratinocytes cells had higher migration rate with peptides. When the bacteria are infected, the cells die and cannot move.
